# Supplementary material for: IDH status dictates oHSV mediated metabolic reprogramming affecting anti-tumor immunity
Source: Nat Commun. 2025 Apr 24;16:3874. doi: 10.1038/s41467-025-58911-2 (PMC12022073; doi:10.1038/s41467-025-58911-2)
Supplement: Supplementary file 1 — Supplementary Information [file 41467_2025_58911_MOESM1_ESM.pdf]

Supplementary Information

IDH status dictates oHSV mediated metabolic reprogramming affecting anti-tumor immunity.

Upasana Sahu[#1,2], Matthew P. Mullarkey[3], Sara A. Murphy[1,2,4], Joshua C. Anderson[5], Vasanta Putluri[6], Abu Hena Mostafa Kamal[6,7], Jun Hyoung Park[8], Tae Jin Lee[9], Alexander L. Ling[10], Benny A. Kaipparettu[8], Ashok Sharma[9], Nagireddy Putluri[6,7], Pamela L. Wenzel[11], Christopher D. Willey[5], E. Antonio Chiocca[10], James M. Markert[12], Balveen Kaur[#1,2].

# Correspondence: bkaur@augusta.edu, usahu@augsuta.edu

Supplementary Figures 1 – 11

Supplementary Tables 1

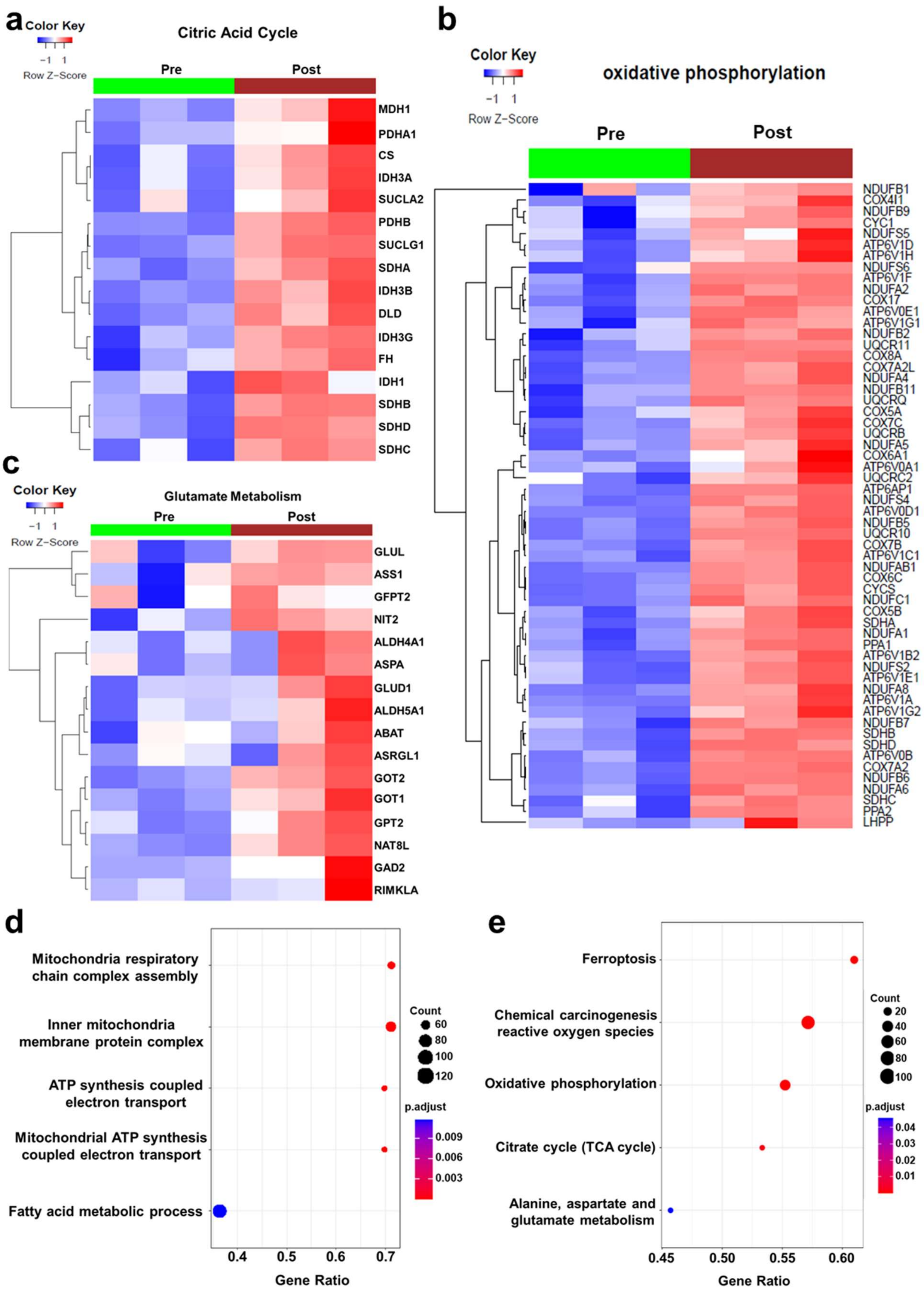
a
Color Key
-1 1
Row Z-Score
Citric Acid Cycle
Pre
Post
MDH1
PDHA1
CS
IDH3A
SUCLA2
PDHB
SUCLG1
SDHA
IDH3B
DLD
IDH3G
FH
IDH1
SDHB
SDHD
SDHC
b
Color Key
-1 1
Row Z-Score
oxidative phosphorylation
Pre
Post
NDUFB1
COX4I1
NDUFB9
CYC1
NDUFS5
ATP6V1D
ATP6V1H
NDUFS6
ATP6V1F
NDUFA2
COX17
ATP6V0E1
ATP6V1G1
NDUFB2
UQCR11
COX8A
COX7A2L
NDUFA4
NDUFB11
UQCRQ
COX5A
COX7C
UQCRB
NDUFA5
COX6A1
ATP6V0A1
UQCRC2
ATP6AP1
NDUFS4
ATP6V0D1
NDUFB5
UQCR10
COX7B
ATP6V1C1
NDUFAB1
COX6C
CYCS
NDUFC1
COX5B
SDHA
NDUFA1
PPA1
ATP6V1B2
NDUFS2
ATP6V1E1
NDUFA8
ATP6V1A
ATP6V1G2
NDUFB7
SDHB
SDHD
ATP6V0B
COX7A2
NDUFB6
NDUFA6
SDHC
PPA2
LHPP
c
Color Key
-1 1
Row Z-Score
Glutamate Metabolism
Pre
Post
GLUL
ASS1
GFPT2
NIT2
ALDH4A1
ASPA
GLUD1
ALDH5A1
ABAT
ASRGL1
GOT2
GOT1
GPT2
NAT8L
GAD2
RIMKLA
d
Mitochondria respiratory chain complex assembly
Inner mitochondria membrane protein complex
ATP synthesis coupled electron transport
Mitochondrial ATP synthesis coupled electron transport
Fatty acid metabolic process
0.4 0.5 0.6 0.7
Gene Ratio
Count
60
80
100
120
p.adjust
0.009
0.006
0.003
e
Ferroptosis
Chemical carcinogenesis reactive oxygen species
Oxidative phosphorylation
Citrate cycle (TCA cycle)
Alanine, aspartate and glutamate metabolism
0.45 0.50 0.55 0.60
Gene Ratio
Count
20
40
60
80
100
p.adjust
0.04
0.03
0.02
0.01

Figure S1. Changes in GSEA pathways reflective of mitochondria bioenergetics [1]. a-c) Heatmap shows relative enrichment of gene signatures involved in the citric acid cycle (a), oxidative phosphorylation (b) and glutamate metabolism (c) in rGBM patients following oHSV G207 treatment (Post). **d-e)** Dot plots of GBM patients' gene ontology (GO) enriched pathways after oHSV G207 treatment from RNA sequencing of the tumor biopsies from GBM patients when comparing pre to post oHSV G207 treatment (n=3).

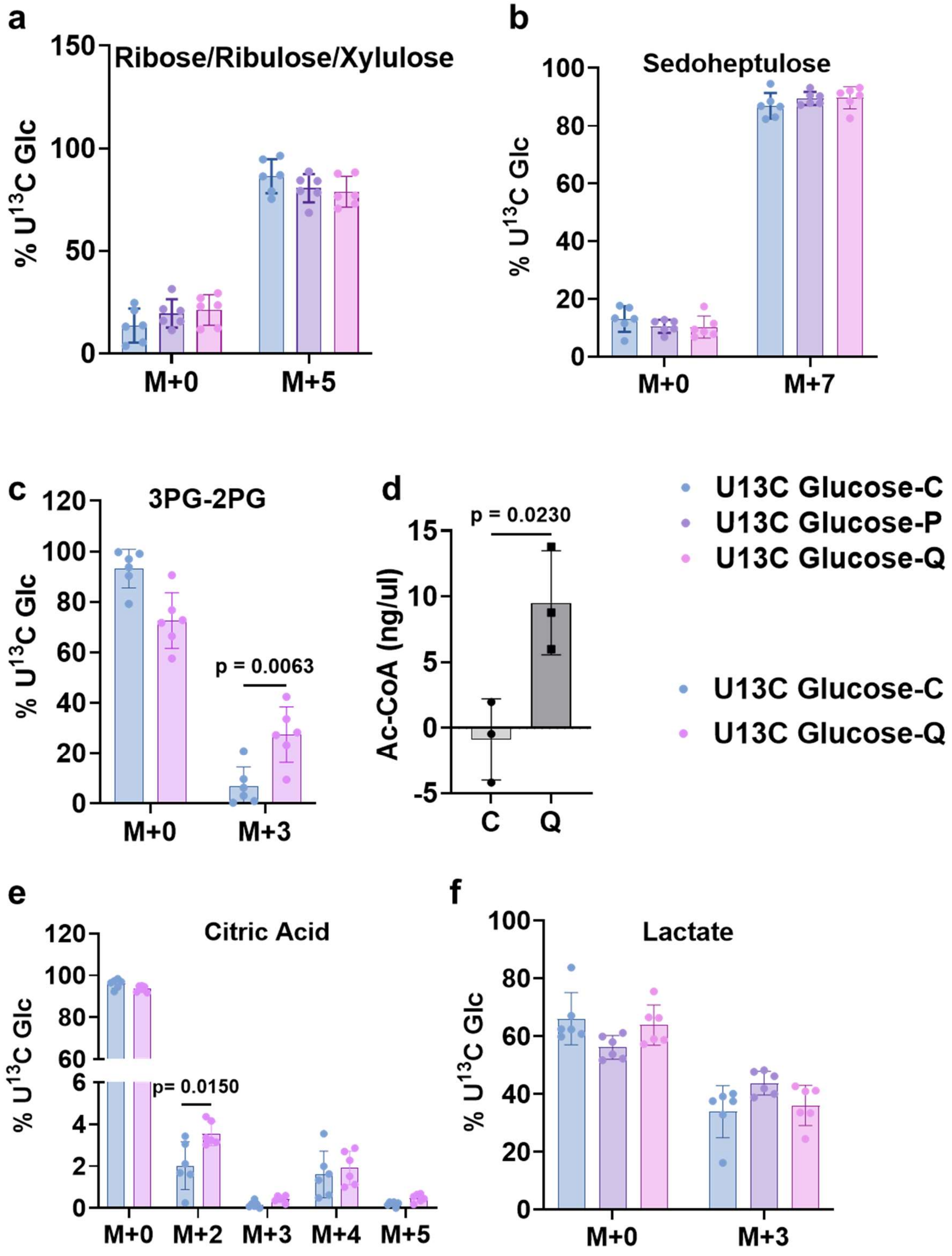

Figure S2. Fate of glucose in oHSV infected GBM cells. Effect of oHSV (oHSVQ (Q) and P) treatment on GBM12 glucose utilization. Briefly GBM12 cells infected with Q or P for 12h were cultured in glucose free media for 4h followed by supplementation with U-13C glucose and 2%

dialyzed FBS for 6 h were analyzed by LC/MS (n =6/group) for the indicated metabolites. **a-b)** Percentage of the indicated ^{13}C labeled metabolites in the Pentose phosphate pathway metabolites: Ribose/Ribulose/Xylulose (A) and Sedoheptulose (B) (n=6). **c)** Percentage of ^{13}C labeled carbons for 3-Phosphoglycerate/2-Phosphoglycerate in Q infected GBM12 cells (n=6). **d) C**oncentration of Acetyl-CoA in control and Q infected GBM12 cells (n=3). **e)** Percentage of ^{13}C labeled carbons for TCA cycle metabolite Citrate in Q infected GBM12 cells (n=6). **f)** Percentage of ^{13}C labeled carbons for Lactate in uninfected and oHSVs Q or P infected GBM12 cells (n=6). Data= Mean ± S.D., n ≥ 3 independent replicates. (Two-way ANOVA, *t* tests). Source data are provided as a Source Data file.

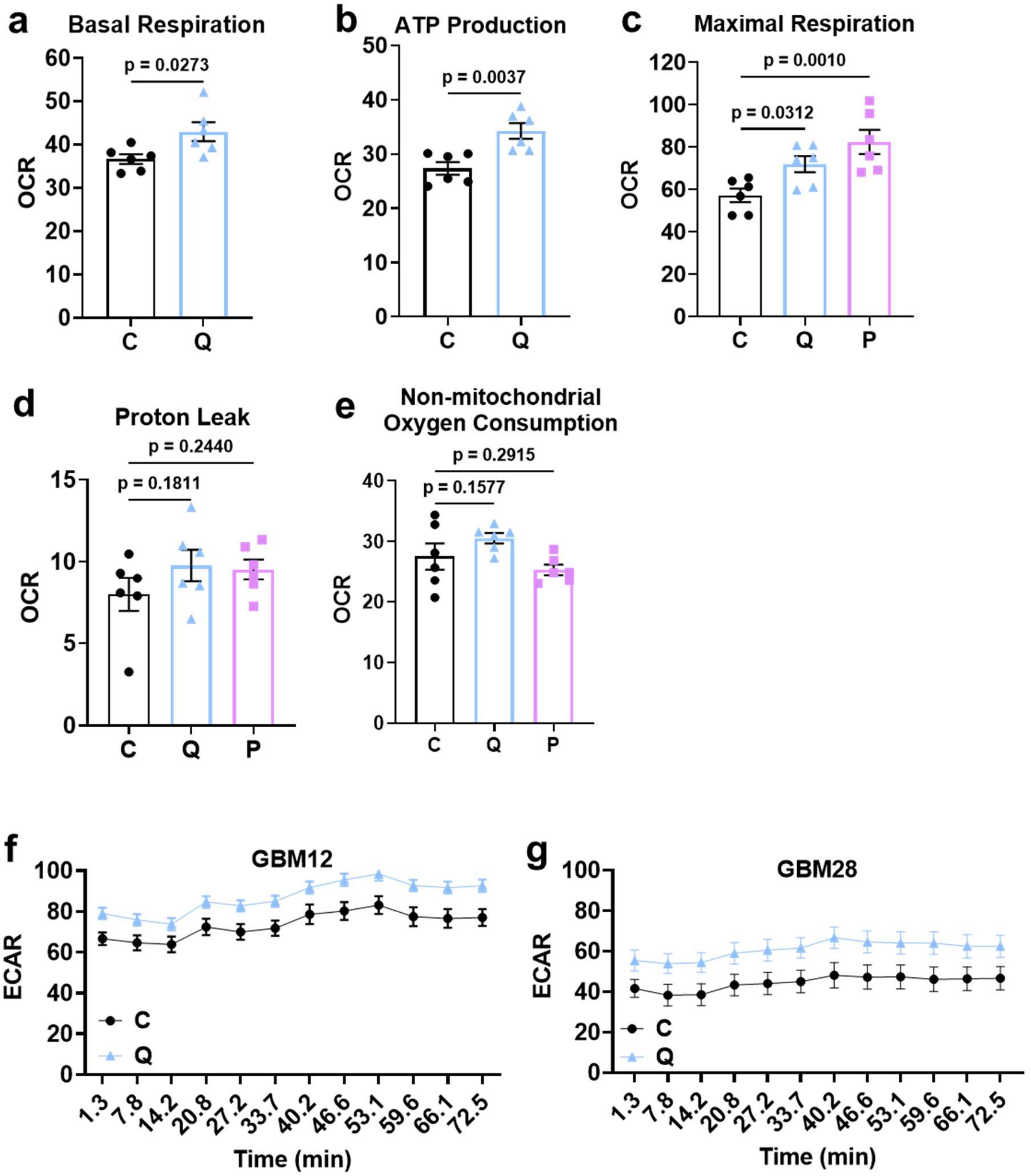

Figure S3. Seahorse analysis of oxygen consumption rate in uninfected and oHSV infected cells. a-b) The rates of basal respiration (a) and ATP production (b) in Q infected and uninfected (C) GBM12 cells (n=6). **c-e)** Maximal respiratory capacity (c), Proton leak (d) and non-mitochondrial oxygen consumption (e) in uninfected and Q or P infected GBM12 cells (n=6). **f-g)** Extracellular acidification rate (ECAR) in Q infected GBM12 cells, n=6 (f) and GBM28 cells, n=3 (g) relative to uninfected control. Data= mean ± S.D. from $n \geq 3$ independent replicates. (One-way ANOVA, unpaired two-tailed Student's t-test). Source data are provided as a Source Data file.

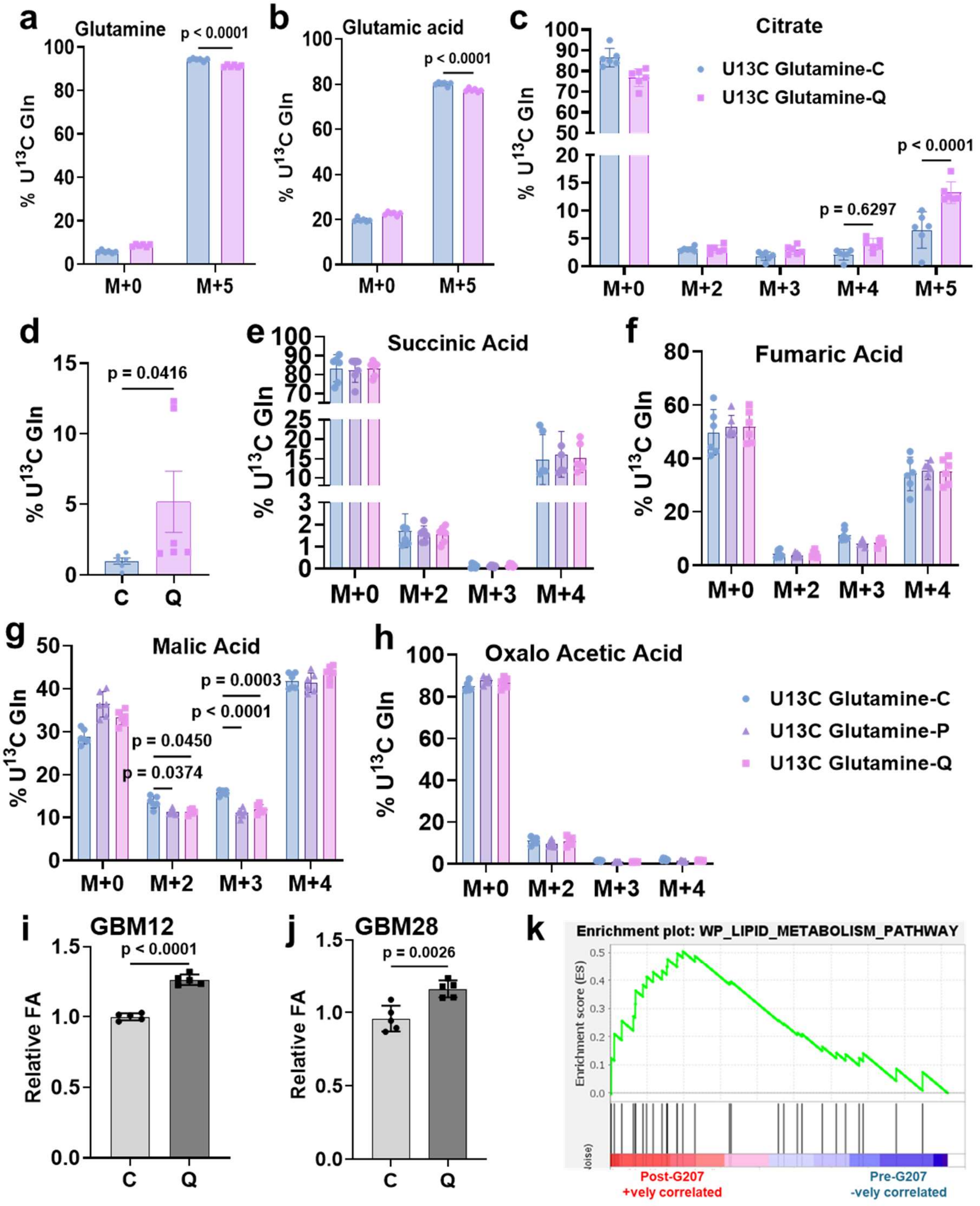

Figure S4. Reductive glutamine metabolism in GBM cells following oHSV infection. oHSV treatment results in enhanced utilization of glutamine by GBM cells metabolizing α-Ketoglutarate through reductive activity of IDH generating citrate, routed via reductive carboxylation. **a-c)** Total percentage of ^{13}C labeled indicated metabolite coming from the utilization of U-13C Glutamine: Glutamine (a), Glutamic acid (b), Citrate (c) in control and Q treated GBM12 cells. **d)** Fold change

in the ratio of M+5 citrate to M+5 α-Ketoglutarate indicating increased portion of citrate derived from α-Ketoglutarate through active reductive carboxylation. **e-h)** Levels of U-13C glutamine labeled succinate (e), fumarate (f) malate (g) and oxaloacetate (h) in C, Q and P treated GBM12 cells (n=6). **i-j)** Relative free fatty acid in C or Q treated GBM12 (i) and GBM28 (j) glioma cells. **k)** GSEA of RNA sequencing data from GBM patients (n=3) before and after oHSV G207 treatment depicting differentially expressed genes involved in the lipid metabolism pathway. Data are shown as mean ± S.D., n=6 for a-h, n=5 for i-j (independent replicates). (Two-way ANOVA, unpaired one-tailed Student's t-test (d), unpaired two-tailed Student's t-test (i-j)). Source data are provided as a Source Data file.

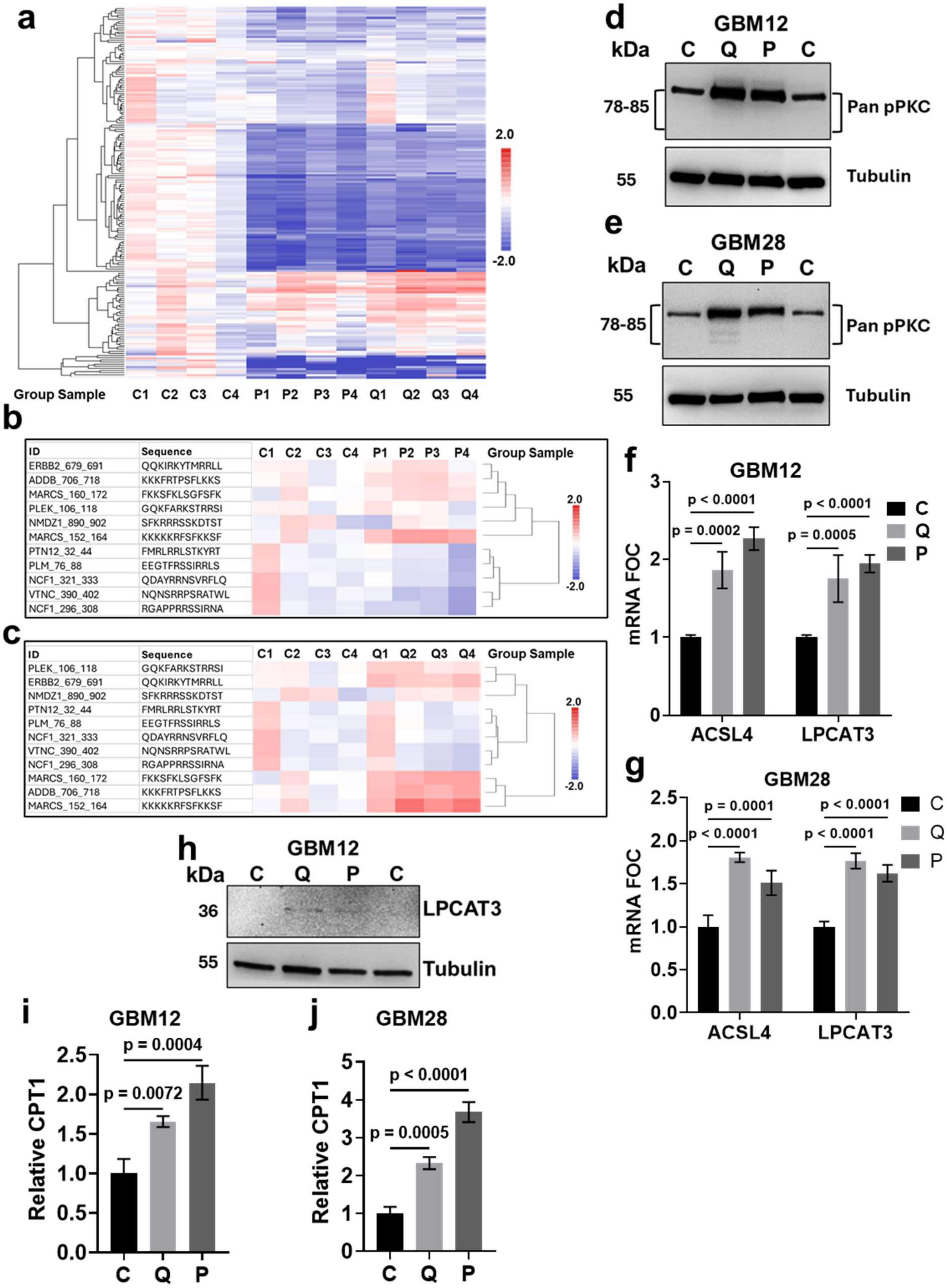
a
Group Sample
C1 C2 C3 C4 P1 P2 P3 P4 Q1 Q2 Q3 Q4
2.0
-2.0
b
ID
Sequence
C1 C2 C3 C4 P1 P2 P3 P4
Group Sample
ERBB2_679_691 QQKIRKYTMRRLL
ADDB_706_718 KKKFRTPSFLKKS
MARCS_160_172 FKKSFKLSGFSFK
PLEK_106_118 GQKFARKSTRRSI
NMDZ1_890_902 SFKRRRSSKDTST
MARCS_152_164 KKKKKRFSFKKSF
PTN12_32_44 FMRLRRLSTKYRT
PLM_76_88 EEGTFRSSIRRLS
NCF1_321_333 QDAYRRNSVRFLQ
VTNC_390_402 NQNSRRPSRATWL
NCF1_296_308 RGAPPRRSSIRNA
2.0
-2.0
c
ID
Sequence
C1 C2 C3 C4 Q1 Q2 Q3 Q4
Group Sample
PLEK_106_118 GQKFARKSTRRSI
ERBB2_679_691 QQKIRKYTMRRLL
NMDZ1_890_902 SFKRRRSSKDTST
PTN12_32_44 FMRLRRLSTKYRT
PLM_76_88 EEGTFRSSIRRLS
NCF1_321_333 QDAYRRNSVRFLQ
VTNC_390_402 NQNSRRPSRATWL
NCF1_296_308 RGAPPRRSSIRNA
MARCS_160_172 FKKSFKLSGFSFK
ADDB_706_718 KKKFRTPSFLKKS
MARCS_152_164 KKKKKRFSFKKSF
2.0
-2.0
d
GBM12
kDa
C Q P C
78-85
Pan pPKC
55
Tubulin
e
GBM28
kDa
C Q P C
78-85
Pan pPKC
55
Tubulin
f
GBM12
mRNA FOC
p < 0.0001
p = 0.0002
p < 0.0001
p = 0.0005
C
Q
P
ACSL4
LPCAT3
g
GBM28
mRNA FOC
p = 0.0001
p < 0.0001
p < 0.0001
p < 0.0001
C
Q
P
ACSL4
LPCAT3
h
GBM12
kDa
C Q P C
36
LPCAT3
55
Tubulin
i
GBM12
Relative CPT1
p = 0.0004
p = 0.0072
C Q P
j
GBM28
Relative CPT1
p < 0.0001
p = 0.0005
C Q P

Figure S5. oHSV infection activates PKC signaling in GBM cells.

a) Phosphopeptide signals were displayed as a heatmap (stratified by change from experimental mean). Samples (column) and peptides (row) were hierarchically clustered with a geometric means distance method, with dendrograms indicating sample similarity. *Red* and *blue* indicate higher and lower phosphorylation, respectively, n=4. **b-c)** Heatmap of PKCα, PKCβ, or PKCᵞ target phosphopeptide signature in P infected (b) and Q infected (c) GBM12 cells compared to uninfected control (*red* indicates higher, and *blue* indicates lower phosphorylation), n=4. **d-e)** GBM cells were infected with Q or P at MOI 0.01 for 48h. Western blot analysis of Phospho-PKC (pan) (βII Ser660) in P and Q infected GBM12 cells (d) and GBM28 cells (e). **f-g)** qPCR analysis for ACSL4 and LPCAT3 in Q or P infected GBM12 cells (f) and GBM28 cells (g) (n=3). **h)** LPCAT3 protein expression in uninfected and Q or P infected GBM12 cells. **i-j)** Levels of CPT1A in Q or P infected cells relative to control by qPCR analysis in GBM12 (i) and GBM28 (j) cells (n=3). Data= mean ± S.D., n ≥ 3 independent replicates. (One-way ANOVA). Tubulin was used as a loading control. Source data are provided as a Source Data file.

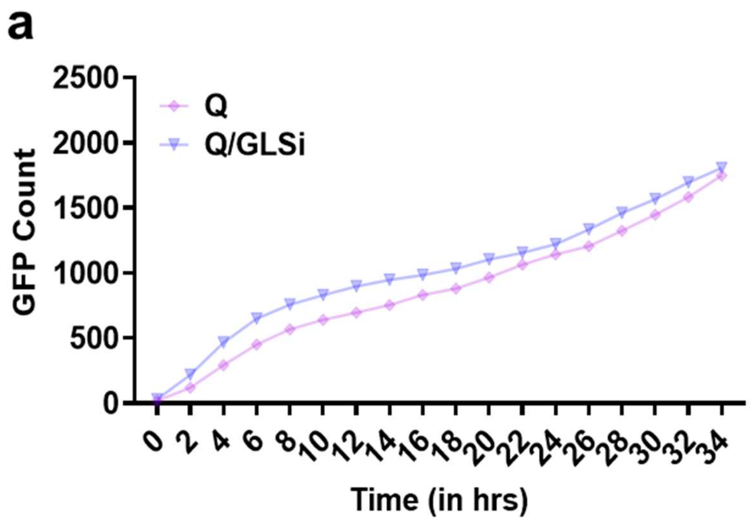

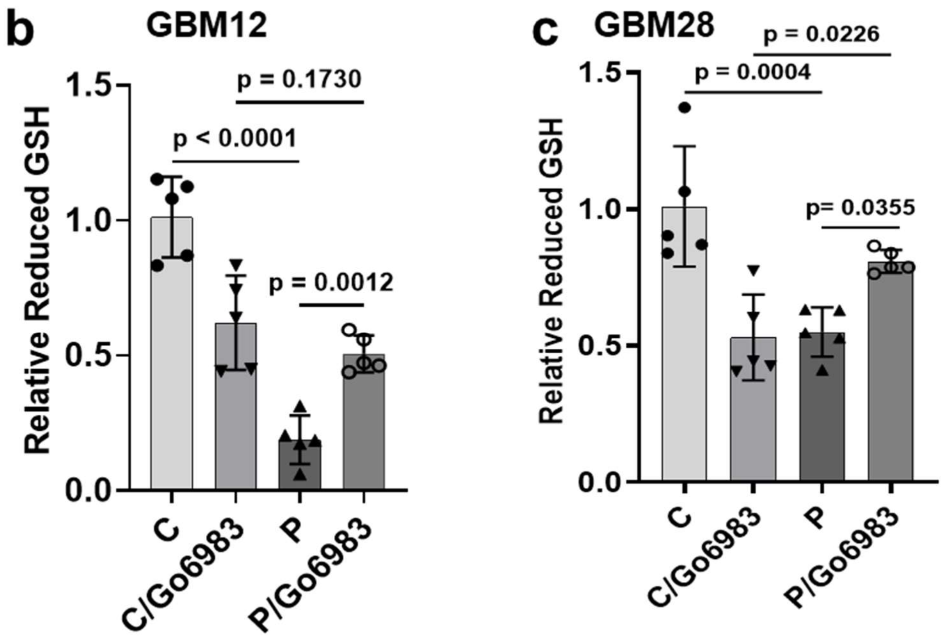

Figure S6. Effect of metabolic inhibitors on virus replication and cellular glutathione levels. **a)** Kinetics of oHSV Q (pink line) and Q + GLSi (light blue line) replication in GBM12 cells (MOI= 0.01) assessed by GFP expression monitored over time using Cytation 5 live imaging system. Data= mean, n=3 technical replicates. **b-c)** Relative reduced glutathione (GSH) levels in P infected GBM12 cells (b) and GBM28 cells (c) in the absence or presence of PKC inhibitor Go6983 (n=5). Data= mean ± S.D., n≥3 independent replicates. (One-way ANOVA). Source data are provided as a Source Data file.

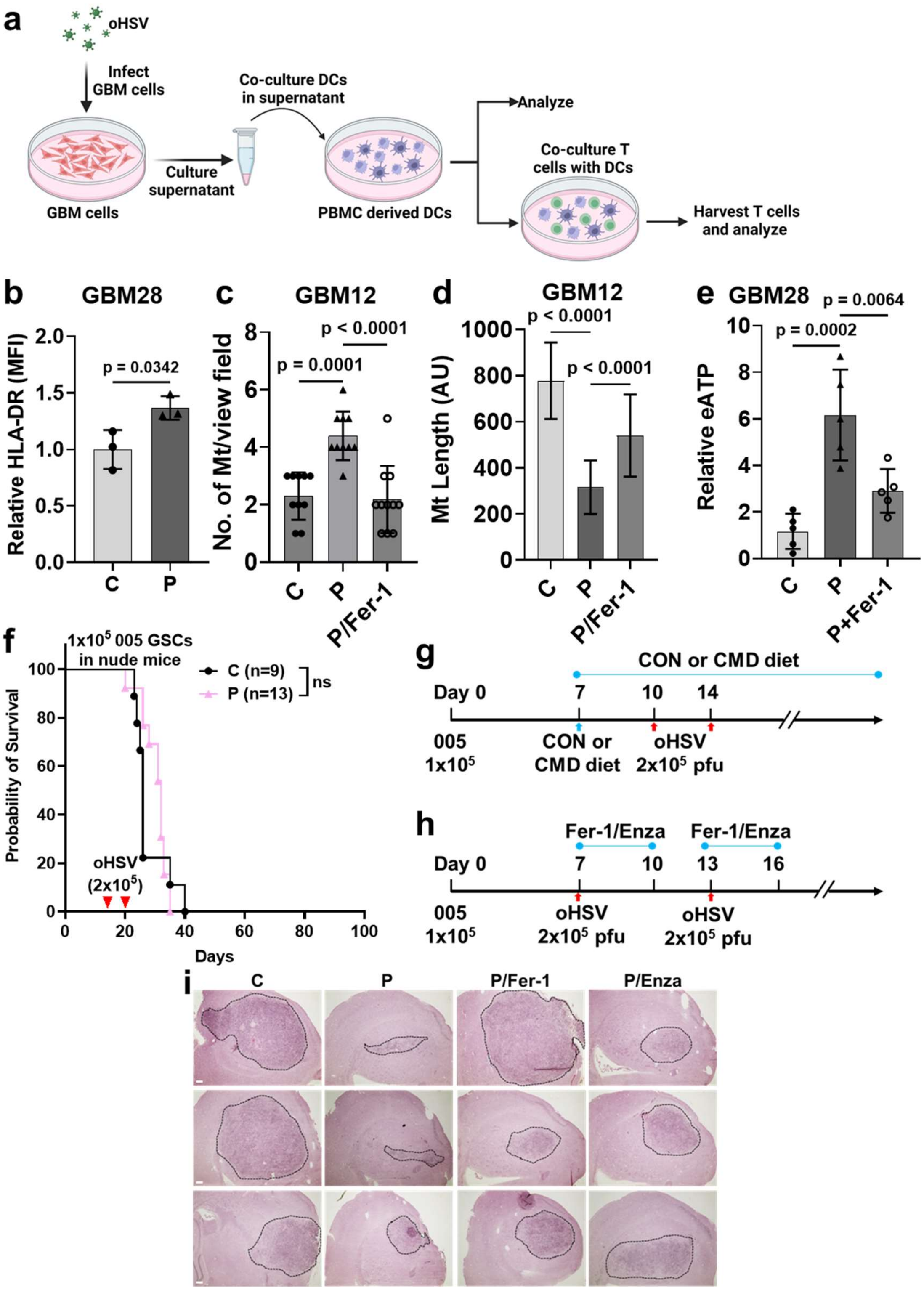

Figure S7. oHSV induced ferroptosis is immunogenic.

a) Experimental setup to assess human PBMC-derived DC maturation and function (activation of T cells) when co-cultured with supernatants from GBM cells infected with P ± Fer-1. [Created in

BioRender. Sahu, U. (2025) https://BioRender.com/y54r719]. **b)** Relative HLA-DR MFI in P infected GBM28 cells compared to uninfected control (n=3). **c-d)** Quantification of TEM images for the number of mitochondria per view field (c) and length of mitochondria (d) in uninfected and P infected GBM12 cells ± Fer-1. **e)** Relative eATP levels of GBM28 cells uninfected and infected with P ± Fer-1 (n=5). **f)** Kaplan-Meier analysis of murine 005 GSC tumor bearing immunocompromised athymic nude mice following treatment with C or P (n = 8 for C group; and n=9 for P group). Values from a single experiment with C and P groups, same in Fig. 7J and Fig. S8F. Log-rank (Mantel-Cox) test. Data= mean ± S.D. **g-h)** Schematic of experimental design for animals on normal or CMD diet (g) and ± Fer-1 or ± Enza (h) *in vivo* experiments. **i)** Hematoxylin and Eosin (H&E) stained brain sections from untreated, P ± Fer-1 or ± Enza treated glioma bearing mice at 4X. Scale bar= 100µm. (n=3 mice/group). (One-way ANOVA, unpaired two-tailed Student's t-test). Source data are provided as a Source Data file.

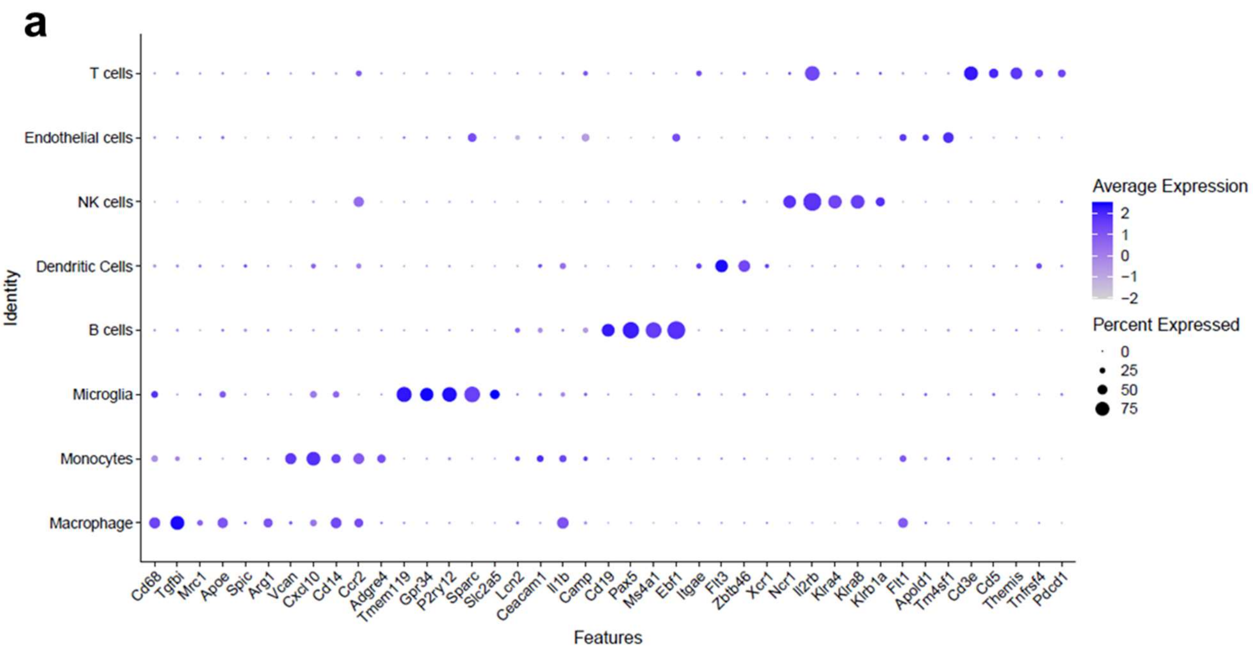

b

	P	P+Fer-1
**B cells**	323	409
**Dendritic cells**	1181	946
**Endothelial cells**	113	186
**Macrophage**	4101	5377
**Microglia**	2185	2541
**Monocytes**	471	566
**Neutrophils**	142	113
**NK cells**	853	782
**T cells**	1135	1507

c

	P	P+Fer-1
**Exausted T cells**	112	152
**Gamma Delta T cells**	36	22
**Gzmk+ CD8 T cells**	115	73
**NaÃ¯ve T**	278	321
**NK cells**	85	156
**Treg**	38	77

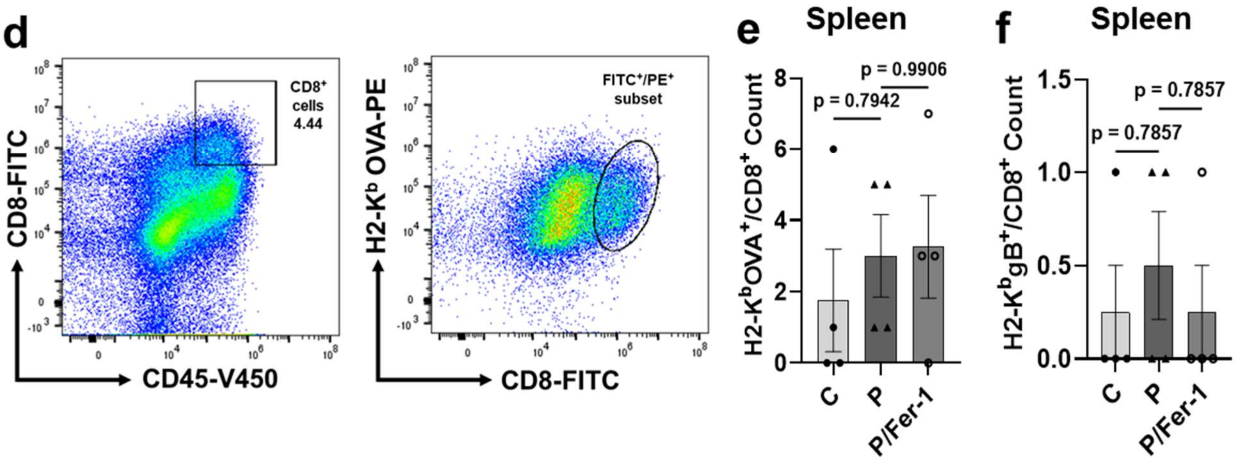

Figure. S8. Effect of ferroptosis on anti-tumor immune response. a) Marker genes used to annotate different cell clusters. Dot size indicates the % of cells of a cluster expressing a given gene, and color intensity represents the level of expression of the gene by that cluster. **b-c)** Tables representing the number of cells in individual cluster for all clusters (b) and T cell subclusters (c) in P and P+Fer-1 treated samples. **d)** Gating strategy for analyzing the tetramer staining. **e-f)** Antitumor- (c) and antiviral- (b) specific T cells were analyzed by OT-1-tetramer and HSV gB tetramer staining in splenocytes at day 21 post-tumor implantation (n=4). (One-way ANOVA). Source data are provided as a Source Data file.

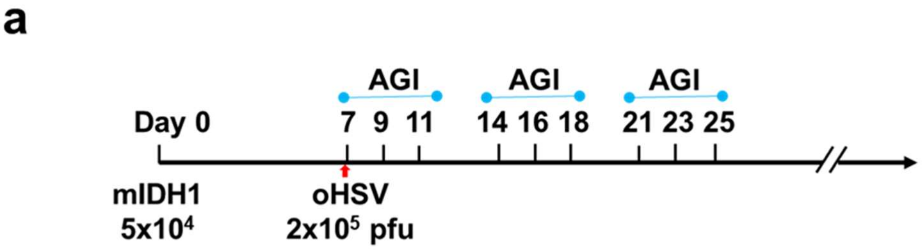

b

Comparison	P values	N values
C (MS 26d) : P (MS 35d)	0.9121	C=10 : P= 20
C (MS 26d) : C/AGI (MS 27.5d)	0.2495	C=10 : C/AGI= 10
C (MS 26d) : P/AGI (MS 40d)	0.1538	C=10 : P/AGI= 18
P (MS 35d) : C/AGI (MS 27.5d)	0.1230	P=20 : C/AGI= 10
P (MS 35d) : P/AGI (MS 40d)	0.1908	P=20 : P/AGI= 18
C/AGI (MS 27.5d) : P/AGI (MS 40d)	**0.0021	C/AGI=10 : P/AGI= 18

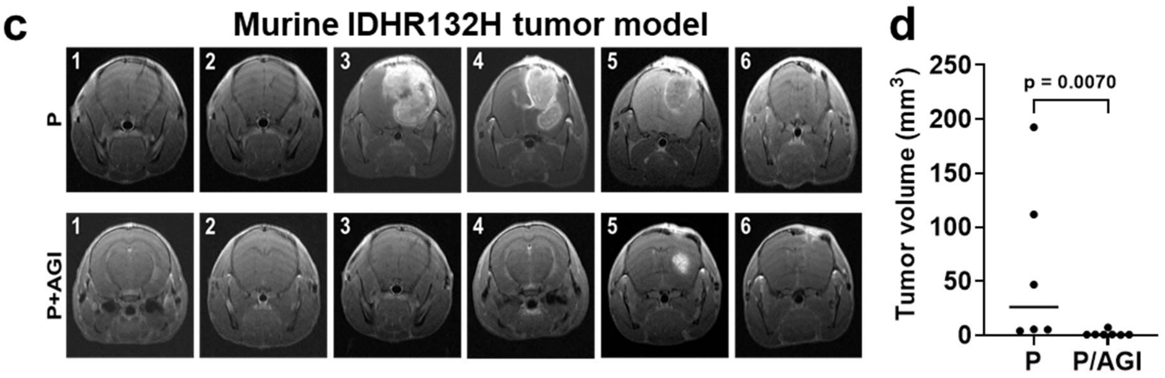

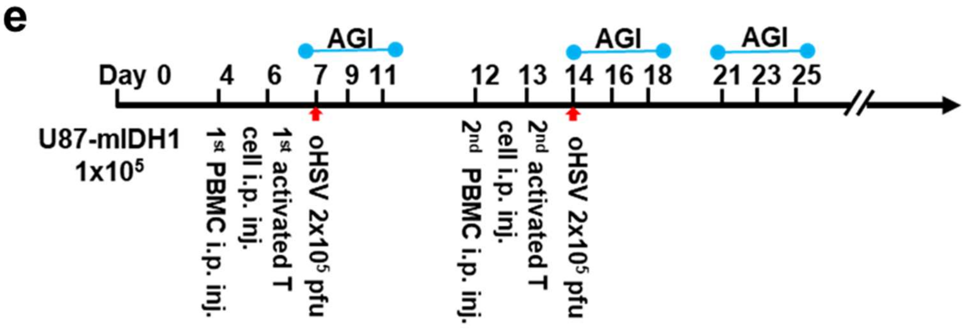

Figure S9. Reductive carboxylation regulates oHSV induced anti-tumor immunity. a) Experimental design to access the survival of IDHR132H glioma-bearing C57BL/6 mice treated with AGI-5198 in combination with oHSV therapy. **b)** Table showing comparison of different

groups indicating median survival (MS), number of mice/group and p-values of IDHR132H glioma-bearing C57BL/6 mice treated with P ± AGI-5198. Log-rank (Mantel-Cox) test. (**P < 0.01). **c-d)** MRI 35 days post tumor implantation (c) and quantification of tumor volume (d) of C57BL/6 mice implanted with murine IDHR132H tumors followed by treatment with P ± AGI-5198. (Mann-Whitney U test; n=6/group). **e)** Experimental design for survival of U87-mIDH glioma bearing NSG mice treated with P ± AGI-5198. Source data are provided as a Source Data file.

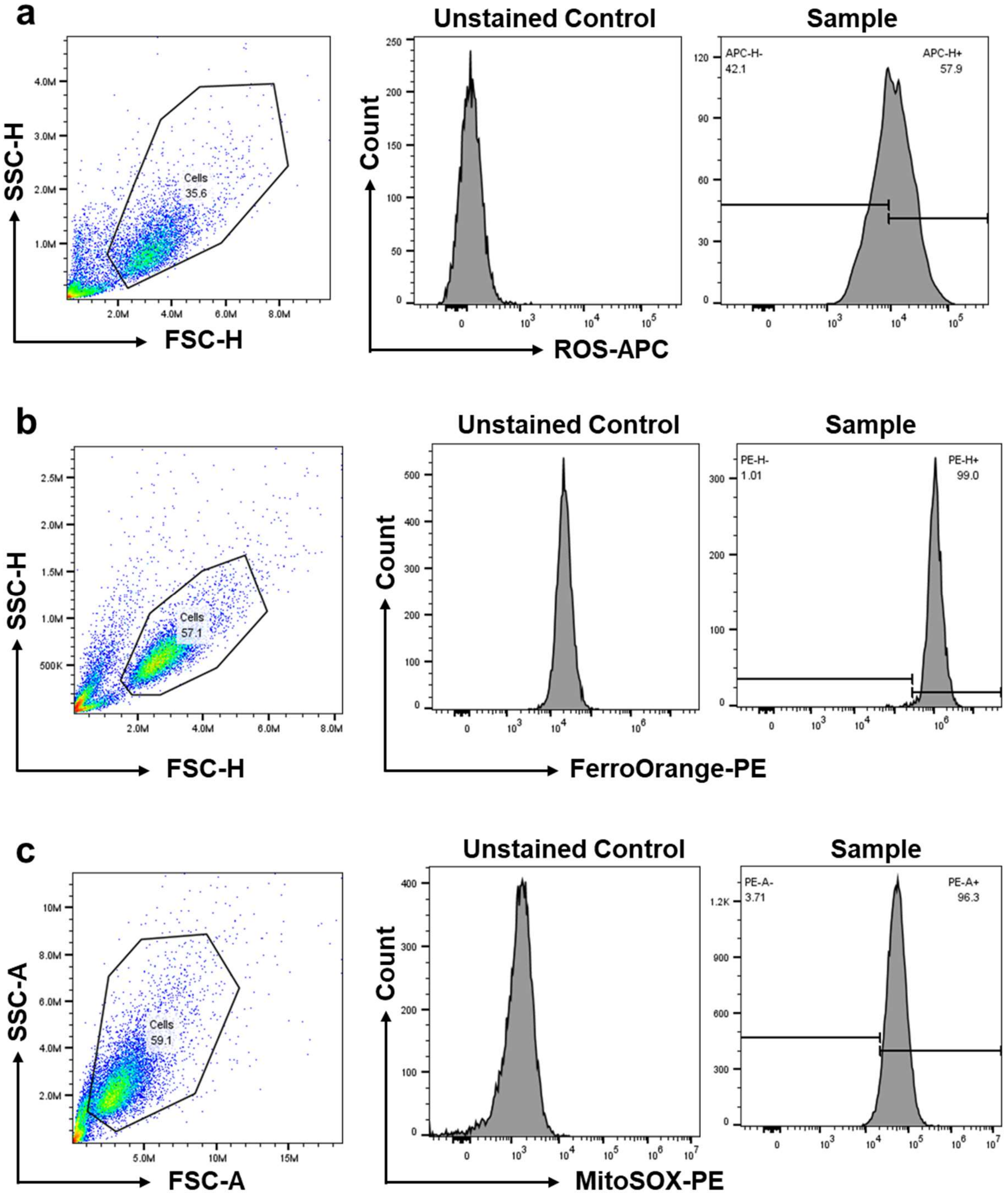

Figure S10. Flow cytometry gating strategies. Gating strategies for flow cytometry analysis of glioma cells for: **a)** cellular ROS corresponding to data depicted in Fig. 5a, Fig. 5d and Fig. 9e-f. **b)** FerroOrange staining corresponding to data shown in Fig. 5m and Fig. 5n, and **c)** mitochondrial ROS by mitoSOX staining corresponding to the data represented in Fig. 5b and Fig. 9g-h. Positive cells were gated based on unstained control cells.

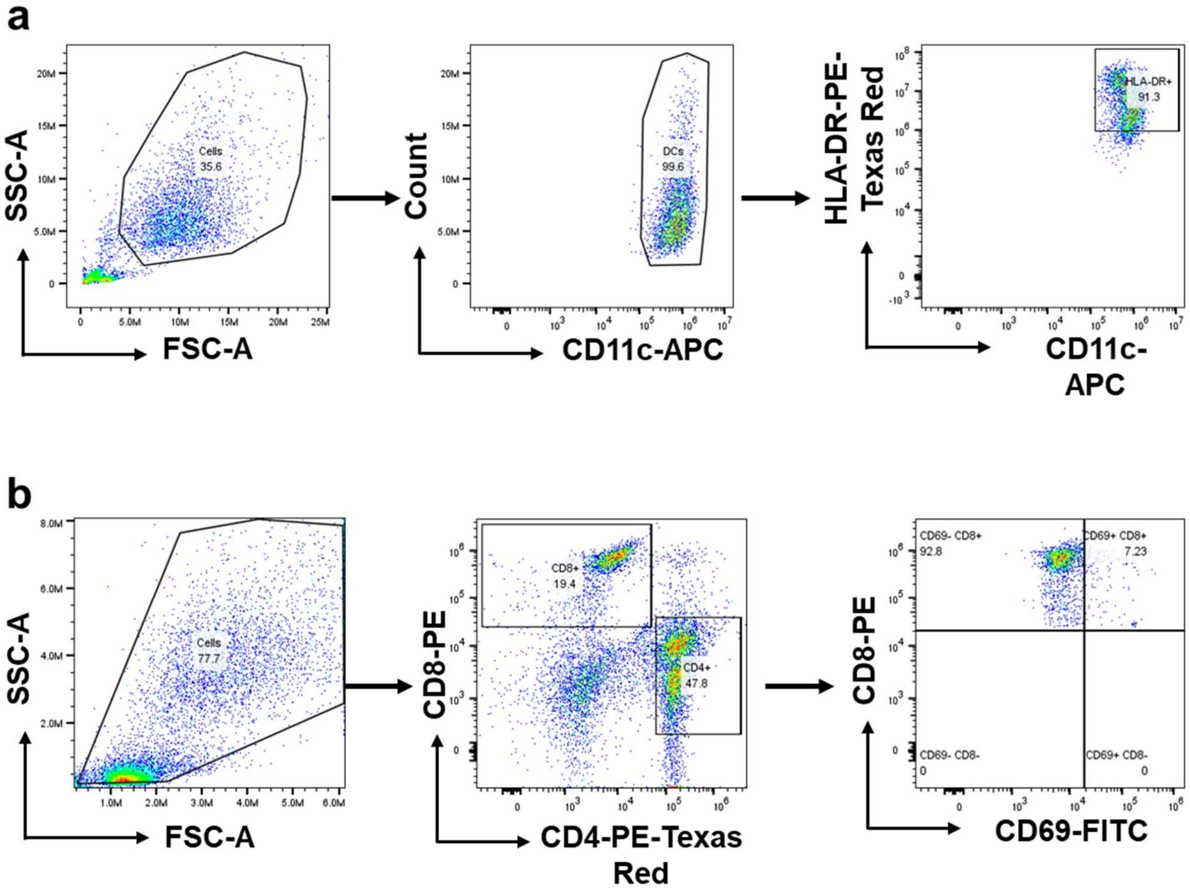

Figure S11. Flow cytometry gating strategies for DC and T cell activation. Gating strategies for flow cytometry analysis of **a)** dendritic cell activation by HLA-DR corresponding to data represented in Fig. 7b, Fig. 7g and Fig. S7b and **b)** T cells activation by CD8+/CD69+ cells corresponding to the data shown in Fig. 7c.

Supplementary Table 1. List of antibodies used for this study.

Antibody	Catalogue number	Vendor	Dilution for western blot	Dilution for IHC	Dilution for Flow Cytometry
ACSL4	MA5-31548	ThermoFisher Scientific	1:1000		
4-HNE	MA5-27570	ThermoFisher Scientific	1:1000		
GPX4	52455S	Cell Signaling Technology	1:1000		
CPT1A	12252S	Cell Signaling Technology	1:1000		
LPCAT3	72964	Cell Signaling Technology	1:500		
Pan-p-PKC (βII S660)	9371	Cell Signaling Technology	1:500		
Beta tubulin	2128L	Cell Signaling Technology	1:3000		
Anti-rabbit IgG	7074	Cell Signaling Technology	1:10000		
Anti-mouse IgG	7076	Cell Signaling Technology	1:10000		
CD8	PA5-88265	ThermoFisher Scientific		1:500	
Anti-rabbit IgG	A48282	ThermoFisher Scientific		1:250	
CD11c	559877	BD Biosciences			5ul/test
HLA-DR	562331	BD Biosciences			1ul/test
CD8	555635	BD Biosciences			5ul/test
CD69	557049	BD Biosciences			1ul/test
CD45	560520	BD Biosciences			1ul/test
CD8	553031	BD Biosciences			1ul/test
SIINFEKL-H2Kb-OVA	chicken ova 257-264	NIH Tetramer Core Facility			3μg/ml
SSIEFARL-H2Kb-gB	HSV-1 gB 498-505	NIH Tetramer Core Facility			2μg/ml

1. Miller, K.E., et al., *Immune Activity and Response Differences of Oncolytic Viral Therapy in Recurrent Glioblastoma: Gene Expression Analyses of a Phase IB Study.* Clin Cancer Res, 2022. **28**(3): p. 498-506.
