# Supplementary material for: IDH status dictates oHSV mediated metabolic reprogramming affecting anti-tumor immunity
Source: Nat Commun. 2025 Apr 24;16:3874. doi: 10.1038/s41467-025-58911-2 (PMC12022073; doi:10.1038/s41467-025-58911-2)
Supplement: Supplementary file 2 — Description of Additional Supplementary Files [file 41467_2025_58911_MOESM2_ESM.pdf]

Description of Additional Supplementary Files

Supplementary Data 1

Data generated from the Stable Isotope-Labeled Glucose Flux.

Supplementary Data 2

Data generated from the Stable Isotope-Labeled Glutamine Flux.

Supplementary Data 3

Data generated from Kinome analysis.
