# Supplementary material for: IDH status dictates oHSV mediated metabolic reprogramming affecting anti-tumor immunity
Source: Nat Commun. 2025 Apr 24;16:3874. doi: 10.1038/s41467-025-58911-2 (PMC12022073; doi:10.1038/s41467-025-58911-2)
Supplement: Supplementary file 6 — Reporting Summary [file 41467_2025_58911_MOESM6_ESM.pdf]

nature portfolio

Corresponding author(s): Balveen Kaur, Upasana Sahu (NCOMMS-24-40537B)

Last updated by author(s): Apr 3, 2025

Reporting Summary

Nature Portfolio wishes to improve the reproducibility of the work that we publish. This form provides structure for consistency and transparency in reporting. For further information on Nature Portfolio policies, see our Editorial Policies and the Editorial Policy Checklist.

Statistics

For all statistical analyses, confirm that the following items are present in the figure legend, table legend, main text, or Methods section.

n/a	Confirmed	
☐	☒	The exact sample size (*n*) for each experimental group/condition, given as a discrete number and unit of measurement
☐	☒	A statement on whether measurements were taken from distinct samples or whether the same sample was measured repeatedly
☐	☒	The statistical test(s) used AND whether they are one- or two-sided<br>*Only common tests should be described solely by name; describe more complex techniques in the Methods section.*
☐	☒	A description of all covariates tested
☐	☒	A description of any assumptions or corrections, such as tests of normality and adjustment for multiple comparisons
☐	☒	A full description of the statistical parameters including central tendency (e.g. means) or other basic estimates (e.g. regression coefficient) AND variation (e.g. standard deviation) or associated estimates of uncertainty (e.g. confidence intervals)
☐	☒	For null hypothesis testing, the test statistic (e.g. *F*, *t*, *r*) with confidence intervals, effect sizes, degrees of freedom and *P* value noted<br>*Give P values as exact values whenever suitable.*
☒	☐	For Bayesian analysis, information on the choice of priors and Markov chain Monte Carlo settings
☒	☐	For hierarchical and complex designs, identification of the appropriate level for tests and full reporting of outcomes
☒	☐	Estimates of effect sizes (e.g. Cohen's *d*, Pearson's *r*), indicating how they were calculated

Our web collection on statistics for biologists contains articles on many of the points above.

Software and code

Policy information about availability of computer code

Data collection	Mitochondrial stress assay data was collected using a Seahorse XFe96 analyzer and software from Agilent Technologies, data plotted using GraphPad Prism. Mass spectrometry data were acquired via multiple reaction monitoring (MRM) using a 6495 Triple Quadrupole mass spectrometry coupled to an HPLC system (Agilent Technologies, Santa Clara, CA) Agilent Mass Hunter Software. Kinomic profiling was done using the PamStation®12 Platform Technology (PamGene, International, Den Bosch, The Netherlands) at UAB Kinome Core. Flow cytometry data were collected on Novocyte Quanteon flow analyzer.
Data analysis	Softwares used for data analysis are described in the 'Methods' section. Statistical analysis was performed using GraphPad Prism v10. Flow cytometry analysis was performed using FlowJo software. Tumor volume analysis was done using ImageJ software. Data analysis for bulk RNA-seq, scRNA-seq, stable isotope labeled metabolite tracing and Kinome profiling is described in the Methods section.

For manuscripts utilizing custom algorithms or software that are central to the research but not yet described in published literature, software must be made available to editors and reviewers. We strongly encourage code deposition in a community repository (e.g. GitHub). See the Nature Portfolio guidelines for submitting code & software for further information.

Data

Policy information about availability of data

All manuscripts must include a data availability statement. This statement should provide the following information, where applicable:

- Accession codes, unique identifiers, or web links for publicly available datasets
- A description of any restrictions on data availability
- For clinical datasets or third party data, please ensure that the statement adheres to our policy

All data are available in the article, supplemental information or source data file. Raw and processed single cell RNA-Seq data from mouse tumors are available at GEO Hub with accession number GSE289317. Uncropped scans of all blots in Figures and Supplementary Figures are provided in the Source Data file. Source data are provided with this paper.

Research involving human participants, their data, or biological material

Policy information about studies with human participants or human data. See also policy information about sex, gender (identity/presentation), and sexual orientation and race, ethnicity and racism.

Reporting on sex and gender	Our study used de-identified published bulk RNA seq data from 2 clinical trials:<br>1. Miller KE, et al. Clin Cancer Res. 2022, PMID: 35105718<br>2. Ling AL, et al. Nature. 2023, PMID: 37853118.
Reporting on race, ethnicity, or other socially relevant groupings	N/A
Population characteristics	N/A
Recruitment	N/A
Ethics oversight	N/A

Note that full information on the approval of the study protocol must also be provided in the manuscript.

Field-specific reporting

Please select the one below that is the best fit for your research. If you are not sure, read the appropriate sections before making your selection.

☒ Life sciences ☐ Behavioural & social sciences ☐ Ecological, evolutionary & environmental sciences

For a reference copy of the document with all sections, see nature.com/documents/nr-reporting-summary-flat.pdf

Life sciences study design

All studies must disclose on these points even when the disclosure is negative.

Sample size	For in vivo studies, sample size was determined based on our published reports with this model (PMID: 30479334, PMID: 35022322, PMID: 36796878). Studies were performed using technical controls and/or biological replicates, based on experiments. At least three replicates per condition were analyzed in all cases. In vivo experiments included at least 3 mice to obtain tissue for in vivo analyses including scRNA-seq and tetramer staining. In case of survival studies, at least 9 mice were included per group.
Data exclusions	No data were excluded from the analyses.
Replication	All replication attempts were reproducible.
Randomization	For in vivo studies, mice were randomly assigned to treatment groups, 7 days post tumor implantation. For in vitro studies, cells were plated at the same time in well plates and then randomized into different treatment groups.
Blinding	Investigators were aware of group allocation during in vivo experiments and not blinded to treatment groups during data collection. However, since data collection was done in an automated way for most of the nodal experiments including stable isotope tracing, kinome analysis, sc-seq data analysis and TEM imaging, blinding is not relevant.

Reporting for specific materials, systems and methods

We require information from authors about some types of materials, experimental systems and methods used in many studies. Here, indicate whether each material, system or method listed is relevant to your study. If you are not sure if a list item applies to your research, read the appropriate section before selecting a response.

Materials & experimental systems

n/a	Involved in the study	
	x	Antibodies
	x	Eukaryotic cell lines
x		Palaeontology and archaeology
	x	Animals and other organisms
x		Clinical data
x		Dual use research of concern
x		Plants

Methods

n/a	Involved in the study	
x		ChIP-seq
	x	Flow cytometry
	x	MRI-based neuroimaging

Antibodies

Antibodies used

ACSL4 ThermoFisher Scientific Cat. No. MA5-31548 WB 1:1000
4-HNE ThermoFisher Scientific Cat. No. MA5-27570 WB 1:1000
GPX4 Cell Signaling Technology Cat. No. 52455S WB 1:1000
CPT1A Cell Signaling Technology Cat. No. 12252S WB 1:1000
LPCAT3 Cell Signaling Technology Cat. No. 72964 WB 1:500
pan-p-PKC (βII S660) Cell Signaling Technology Cat. No. 9371 WB 1:500
Beta tubulin Cell Signaling Technology Cat. No. 2128L WB 1:3000
Anti-rabbit IgG Cell Signaling Technology Cat. No. 7074 WB 1:10000
Anti-mouse IgG Cell Signaling Technology Cat. No. 7076 WB 1:10000
CD8 ThermoFisher Scientific Cat. No. PA5-88265 IF 1:500
Anti-rabbit IgG ThermoFisher Scientific Cat. No. A48282 IF 1:250
CD11c BD Biosciences Cat. No. 559877 Flow (5ul/test)
HLA-DR BD Biosciences Cat. No. 562331 Flow (1ul/test)
CD8 BD Biosciences Cat. No. 555635 Flow (5ul/test)
CD69 BD Biosciences Cat. No. 557049 Flow (1ul/test)
CD45 BD Biosciences Cat. No. 560520 Flow (Tetramer staining) (1ul/test)
CD8 BD Biosciences Cat. No. 553031 Flow (Tetramer staining) (1ul/test)

Validation

All antibodies are from commercial sources and have been validated as per previous publications and manufacturer's information.

Eukaryotic cell lines

Policy information about cell lines and Sex and Gender in Research

Cell line source(s)

U87 and U87-mIDH cells and Vero cells were purchased from ATCC. Murine wtIDH and IDHR132H glioma cells were obtained from Dr. Mario Castro's Lab at Michigan University. Human GBM cells were obtained from Dr. Jann N. Sarkaria at Mayo Clinic. 005 cells were obtained from Dr. Inder Verma's Lab at the Salk Institute for Biological Studies in La Jolla.

Authentication

GBM12 and GBM28 cell lines were authenticated by STR profiling at University of Arizona Genetics Core.

Mycoplasma contamination

All cells were checked routinely and tested negative for Mycoplasma.

Commonly misidentified lines (See ICLAC register)

No commonly misidentified cell lines were used in this study.

Animals and other research organisms

Policy information about studies involving animals; ARRIVE guidelines recommended for reporting animal research, and Sex and Gender in Research

Laboratory animals

Animals used were C57BL/6, NSG or Athymic nude adult mice, 6-8 weeks old at the time of tumor implantation. All mice were housed in the mouse pathogen-free barrier facility with free access to food and water at controlled temperature (21±2°C), humidity (55-60%), 12h light/dark cycle approved by the Augusta University Institutional Animal Care and Use Committee (IACUC) (Protocol # 2022-1080).

Wild animals

The study did not involve wild animals.

Reporting on sex

All survival studies were done using both male and female mice. Only female mice were used for tetramer staining , sc-seq and U87-mIDH xenograft model injected with human PBMCs.

Field-collected samples

The study did not involve field-collected samples.

Ethics oversight

All experimental procedures involving mice were reviewed and approved by the Augusta University Institutional Animal Care and Use Committee (IACUC) (Protocol # 2022-1080).

Note that full information on the approval of the study protocol must also be provided in the manuscript.

Plants

Seed stocks	No plants involved in this study.
Novel plant genotypes	N/A
Authentication	N/A

Flow Cytometry

Plots

Confirm that:

- [x] The axis labels state the marker and fluorochrome used (e.g. CD4-FITC).
- [x] The axis scales are clearly visible. Include numbers along axes only for bottom left plot of group (a 'group' is an analysis of identical markers).
- [x] All plots are contour plots with outliers or pseudocolor plots.
- [x] A numerical value for number of cells or percentage (with statistics) is provided.

Methodology

Sample preparation	Sample preparation for ROS, FerroOrange, Tetramer staining, DC and T cell activation are described in the "Methods" section
Instrument	Data were collected using Novocyte Quanteon flow analyzer.
Software	Data were analyzed using FlowJo v10 software.
Cell population abundance	We did not sort the cells for this study.
Gating strategy	Forward vs side scatter was used to identify the cells population of interest and exclude the dead cells and debris. Positive cells were gated based on gating of unstained and single stained cells using appropriate channels. Gating strategy provided in Supplementary Figure 8B.

- [x] Tick this box to confirm that a figure exemplifying the gating strategy is provided in the Supplementary Information.

Magnetic resonance imaging

Experimental design

Design type	Tumor imaging in orthotopic GBM model in syngeneic and immune compromised mice.
Design specifications	T1 weight enhancement scans were acquired to detect tumor sizes at day 35 post tumor implantation.
Behavioral performance measures	N/A

Acquisition

Imaging type(s)	T1W Structural MRI
Field strength	7 Tesla
Sequence & imaging parameters	Multi-slice T1-weighted spin echo images were obtained in the coronal orientation using a repetition time of 1500 ms, echo time of 8 ms and imaging matrix of 256×256 with the field of view of 19.2 × 19.2 mm2, 21 slices per brain volume (slice thickness of 0.75mm).
Area of acquisition	Multiple slices were taken to cover the whole brain

Diffusion MRI ☐ Used ☒ Not used

Preprocessing

Preprocessing software	Paravision (PV6)
Normalization	N/A
Normalization template	N/A
Noise and artifact removal	N/A
Volume censoring	We use ImageJ software for volume analysis.

Statistical modeling & inference

Model type and settings	We performed univariate analysis on the relative tumor volume. Relative tumor volume was calculated by mutiplying slice thickness with the sum of area across the slices.
Effect(s) tested	N/A

Specify type of analysis: ☐ Whole brain ☒ ROI-based ☐ Both

Anatomical location(s): Region of interest (ROI) was drawn manually on the mentioned regions across the slices with visible tumor and tumor volume was calculated using ImageJ software by (sum of area across the slices) x slice thickness (0.75mm).

Statistic type for inference (See Eklund et al. 2016)	N/A
Correction	No correction methods were used.

Models & analysis

n/a	Involved in the study
☒	☐ Functional and/or effective connectivity
☒	☐ Graph analysis
☒	☐ Multivariate modeling or predictive analysis
